# The Use of Physician-Patient Email: A Follow-up Examination of Adoption and Best-Practice Adherence 2005-2008

**DOI:** 10.2196/jmir.1578

**Published:** 2011-02-25

**Authors:** Nir Menachemi, Charles T Prickett, Robert G Brooks

**Affiliations:** ^2^College of MedicineUniversity of South FloridaTampa, FLUnited States; ^1^School of Public HealthUniversity of Alabama at BirminghamBirmingham, ALUnited States

**Keywords:** email, physician-patient relationship

## Abstract

**Background:**

Improved communication from physician- patient emailing is an important element of patient centeredness. Physician-patient email use has been low; and previous data from Florida suggest that physicians who email with patients rarely implement best-practice guidelines designed to protect physicians and patients.

**Objective:**

Our objective was to examine whether email use with patients has changed over time (2005-2008) by using two surveys of Florida physicians, and to determine whether physicians have more readily embraced the best-practice guidelines in 2008 versus 2005. Lastly, we explored the 2008 factors associated with email use with patients and determined whether these factors changed relative to 2005.

**Methods:**

Our pooled time-series design used results from a 2005 survey (targeting 14,921 physicians) and a separate 2008 survey (targeting 7003 different physicians). In both years, physicians practicing in the outpatient setting were targeted with proportionally identical sampling strategies. Combined data from questions focusing on email use were analyzed using chi-square analysis, Fisher exact test, and logistic regression.

**Results:**

A combined 6260 responses were available for analyses, representing a participation rate of 28.2% (4203/14,921) in 2005 and 29.4% (2057/7003) in 2008. Relative to 2005, respondents in 2008 were more likely to indicate that they personally used email with patients (690/4148, 16.6% vs 408/2001, 20.4%, c^2^_1_ = 13.0, *P* < .001). However, physicians who reported frequently using email with patients did not change from 2005 to 2008 (2.9% vs 59/2001, 2.9%). Interest among physicians in future email use with patients was lower in 2008 (58.4% vs 52.8%, c^2^_2_ = 16.6, *P* < .001). Adherence to email best practices remained low in 2008. When comparing 2005 and 2008 adherences with each of the individual guidelines, rates decreased over time in each category and were significantly lower for 4 of the 13 guidelines. Physician characteristics in 2008 that predicted email use with patients were different from 2005. Specifically, in multivariate analysis female physicians (OR 1.48, 95% CI 1.12-1.95), specialist physicians (OR 1.43, 95% CI 1.12-1.84), and those in a multispecialty practice (OR 1.76, 95% CI 1.30-2.37) were more likely than their counterparts to email with patients. Additionally, self-reported computer competency levels (on a 5-point Likert scale) among physicians predicted email use at every level of response.

**Conclusions:**

Email use between physicians and patients has changed little between 2005 and 2008. However, future physician interest in using email with patients has decreased. More troubling is the decrease in adherence to best practices designed to protect physicians and patients when using email. Policy makers wanting to harness the potential benefits of physician-patient email should devise plans to encourage adherence to best practices. These plans should also educate physicians on the existence of best practices and methods to incorporate these guidelines into routine workflows.

## Introduction

The use of email is poised to revolutionize the delivery of health care with improved efficiency, convenience, satisfaction, or access to care [1-4]. In the clinical setting, email has the potential to be a tool of efficiency for physicians and convenience for patients. It offers yet another means of communication for physician and patient, and has even been used by some as a substitute for clinic visits when appropriate [5,6]. Despite the opportunities offered by this communication technology, physicians’ adoption of email with patients remains low [6-10].

Among the current literature, relatively little attention has been given to how physician-patient interaction through email has changed over time. We do know that email usage with health care providers among patients in the general US population continues to increase, albeit slowly [11]. Although early research reported a reluctance by patients to use email as a communication medium with their physicians [10,12], more recent studies have shown patients to be mostly willing to embrace the idea [13-17]. In examining the barriers and facilitators to physician-patient email communication, recent studies have suggested patient age [18,19], patient race [18,19], patient health status [18], physicians’ satisfaction with their work [20], physician specialty [21], and physician workload [22] to be correlated with email usage. Despite the presence of a few scholarly explorations within this niche, more studies are necessary to determine specific aspects of physician adoption of email as a communication medium.

A 2005 study from our group [9] identified correlates of physicians’ adoption of email use with patients and evaluated physician compliance with best-practice recommendations established by the American Medical Association (AMA) and American Medical Informatics Association (AMIA) [23]. We found that certain physician characteristics were associated with increased likelihood of email use with patients. Moreover, we found that the best-practice guidelines designed to protect physicians from liability issues, as well as protecting the privacy of the patient, were being used very infrequently. In the current study, we made ready use of updated physician data that we collected in 2008 from Florida using similar survey techniques. We examined whether email use with patients has changed over time (ie, 2005-2008). Moreover, given that adherence to best practices was low in 2005, we were interested in determining whether physicians more readily embraced the AMA/AMIA guidelines in 2008. Lastly, we explored the current physician and practice characteristics associated with email use with patients; and determined whether these factors changed relative to 2005.

## Methods

We used a pooled time-series design that took advantage of two large-scale surveys of physician use of health information technologies in Florida. The two surveys used similar sampling strategies but did not necessarily target the same physicians in both 2005 and 2008. Data and methods from the first survey, conducted in 2005 (N = 14,921), have been previously reported [24-27]. The second survey, conducted in 2008 (N = 7003), had many identical questions. In the current analysis, we focused upon the questions pertaining to email use that were identical in both surveys and analyzed the combined data. The 2008 survey is attached (see Multimedia Appendix 1).

### Survey

 Similar to the 2005 survey, for the 2008 survey we identified physicians by using Florida Department of Health lists of individuals licensed to practice allopathic or osteopathic medicine and who had a practice address in the state. The focus of the study was on physicians practicing in outpatient settings, so physicians who are typically hospital based (eg, radiologists, pathologists, anesthesiologists, and emergency physicians) were excluded. In 2008, we targeted 50% of all primary care physicians (general internists, family physicians, general pediatricians, general practitioners, and obstetricians/gynecologists) and a 12.5% random stratified sample of other medical and surgical specialists throughout the state. This sampling methodology was proportionately equal to 2005 but sampled half as many physicians.

As in 2005, the 2008 survey was administered with the assistance of an on-campus survey research laboratory that tracked respondents using a 6-digit identifying code. Physicians were initially sent a survey and cover letter describing the study and urging their participation. After 4 weeks, nonrespondents were sent another copy of the survey and an updated cover letter further encouraging their participation. Participants returned their completed surveys in an enclosed prepaid business reply envelope. Staff at the survey research lab kept track of outgoing and incoming surveys and updated addresses retuned as undeliverable as needed. Staff at the survey research lab entered the data and randomly checked for accuracy. The response rate for the 2008 survey was 29.4% (2057/7003), which was very similar to the 2005 response rate of 28.2% (4203/14,921). The institutional review board at Florida State University approved the study protocol.

### Statistical Analyses

Data from 2008 and 2005 were stacked into a single dataset and prepared for analyses. Descriptive statistics were computed for the 2008 sample and various analyses were conducted as follows. First, we compared the frequency of email use among physicians in 2005 with 2008 using chi-square analysis. Next, we compared 2005 and 2008 adherence rates with best-practice email guidelines developed the AMA and AMIA using the Fisher exact test for binary categorical variables. Lastly, based on the 2008 data, we investigated the physician and practice characteristics associated with email use by specifying a logistic regression model that computed odds ratios and 95% confidence intervals. Our predictive model, with email use as the dependent variable, included independent variables for physician gender, age, practice size (measured as the number of physician employed by the practice), physician specialty (primary care or other), practice setting (single or multispecialty), and physician competency as a computer user (measured on a self-reported 5-point Likert scale). This analysis was similar to the one previously conducted with 2005 data [9] to allow for an examination of how current predictors of email use compared with previous findings. All analyses were conducted in SPSS version 16.0 (IBM Corporation, Somers, NY, USA) and significance was considered at the *P* < .05 level.

## Results

A total of 2057/7003 responses were returned in the 2008 survey, representing a 29.4% participation rate. Demographic and practice characteristics of respondents from both 2005 and 2008 are shown in Table 1. Overall, the 2008 sample included a greater proportion of female physicians and a higher proportion of family physicians and general internists. The 2008 sample also included a smaller proportion of surgical and medical specialists. Lastly, respondents in 2008 indicated having greater access to the Internet via high-speed connections, and fewer respondents indicated having dial-up access only.

**Table 1:** Demographic and practice characteristics of responding physicians

		2008 Results (n = 2057)	2005 Results (n = 4203)	*P*-value
Gender: male, n (%)		1434 (70.4%)	2479 (75.9%)	<.001
Mean (range) years in current community		15.0 (<1-53)	14.7 (<1-52)	.14
Mean (range) years since medical school graduation		21.9 (<1-60)	21.3 (<1-65)	.08
**Specialty, n (%)**				
	Family medicine	575 (28.1%)	756 (18.3%)	<.001
	Internal medicine	453 (22.2%)	783 (18.9%)	
	Pediatrics	306 (15.0%)	602 (14.6%)	
	Obstetrics/gynecology	205 (10.0%)	454 (11.0%)	
	General surgery	24 (1.2%)	42 (1.0%)	
	Surgical specialty	154 (7.5%)	393 (9.5%)	
	Medical specialty	184 (9.0%)	709 (17.1%)	
	Other	142 (6.9%)	397 (9.6%)	
Presence of Internet access		1941 (95.5%)	3824 (96.4%)	.07
High-speed access/wireless access		1641 (90.2%)	2857 (85.3%)	<.001
Dial-up connection only		48 (2.6%)	406 (12.1%)	<.001

### Changes in Email Use Over Time

In 2008, 408/2001 physicians (20.4%) indicated that they personally used email from their office to communicate with patients, which was significantly higher than the 16.6% (690/4148) of respondents in 2005 (*P* < .001) (see Table 2). Of those who emailed with patients in 2008, few reported using email frequently (59/408, 14.6%) compared with those who reported using email occasionally (161/408, 40.0%) or rarely (183/408, 45.4%). For those physicians who did use email with their patients, the *frequency* of email use did not differ between 2005 and 2008. Specifically, the 59 doctors in 2008 who indicated that they frequently used email with patients represented 2.9% of a total of 2001 physicians who responded to the email question in the survey. This rate was identical to the 2005 rate reported previously by our group [9].

**Table 2:** Physician’s self-reported email use with patients and other entities

		n (%) of Physicians		c^2^	DF^a^	P-value
		2005 (n = 4148)	2008 (n = 2001)			
Personally uses email with patients from office practice		690 (16.6%)	408 (20.4%)	13.0	1	<.001
**Frequency of email communication with patients**						.41
	Often	120 (17.4%)	59 (14.6%)	1.8	2	
	Occasionally	255 (37.0%)	161 (40.0%)			
	Rarely	314 (45.6%)	183 (45.4%)			
**Would you like to email with patients in the future**						
	Yes	463 (13.4%)	151 (10.1%)	16.6	2	<.001
	No	1823 (52.8%)	869 (58.4%)			
	Do not know yet	1166 (33.8%)	468 (31.5%)			
	Uses email from office practice with entities other than patients	2593 (63.0%)	1272 (63.8%)	0.30	1	.59
**If so, with which groups (check all that apply)?**						
	Family member or caregiver of patients	435 (16.8%)	217 (17.2%)	0.75	1	.75
	Other doctors	1652 (63.8%)	761 (60.2%)	4.6	1	.033
	Business-related communications	1298 (50.1%)	664 (52.5%)	1.9	1	.17
	Hospitals	757 (29.2%)	445 (35.3%)	14.5	1	<.001
	Pharmaceutical companies	531 (20.5%)	304 (24.1%)	6.4	1	.012
	Personal friends or family members	1923 (74.2%)	916 (72.5%)	1.4	1	.24
	Other	333 (12.9%)	130 (10.4%)	5.0	1	.026

^a^ DF: degrees of freedom.

Those who did not currently use email with their patients were asked about their future interest in doing so. Compared with 2005, a greater proportion of physicians in 2008 indicated not being interested in future email use with patients (869/1488, 58.4% in 2008 vs 1823/3452, 52.8% in 2005 in 2008, c^2^_2_ = 16.6, *P* < .001); likewise, the proportion of physician indicating wanting to email with patients in the future decreased over time (463/3452, 13.4% in 2005 vs 151/1488, 10.1% in 2008, c^2^_2_ = 16.6, *P* < .001) (see Table 2). 

The rate of email use by respondents with entities other than patients did not change over time (2593/4148, 63.0% vs 1272/2001, 63.8%, *P* = .59) (Table 2). However, the frequency of email use with specific (nonpatient) groups differed between 2005 and 2008. Specifically, respondents were less likely to email with other doctors in 2008 (1652/2593, 63.8% vs 761/1272, 60.2%, c^2^_1_ = 4.6, *P* = .03), and more likely to email with hospitals (757/2593, 29.2% vs 445/1272, 35.3%; c^2^_1_ = 14.5, *P* < .001) and pharmaceutical companies (531/2593, 20.5% vs 304/1272, 24.1%, c^2^_1_ = 6.4, *P* = .01).

**Figure 1:**
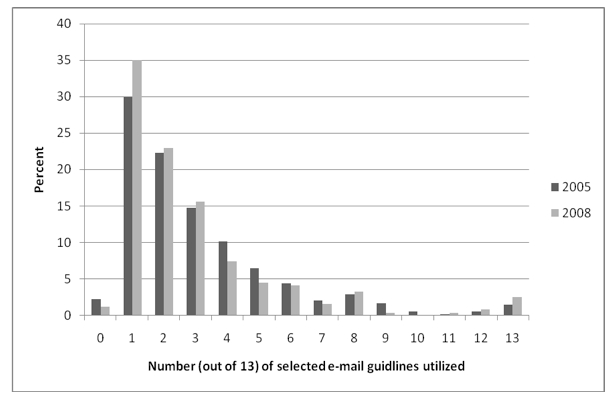
Number and percentage of selected email guideline items being adhered to by physicians in Florida in 2005 and 2008

Of the 408 respondents who indicated using email with patients in 2008, only 6 doctors (1.5%) reported that they abided by all the AMA/AMIA guidelines. Figure 1 presents the number and percentage of guidelines that physicians reported adherence to in 2005 and 2008. While more physicians in 2008 indicating abiding by at least one of the guidelines, the differences overall did not differ. 

When comparing the individual best-practice categories between 2005 and 2008, rates decreased over time in each category and were significantly lower in 2008 for 4 of the 13 guidelines (Table 3).

Specifically, from 2005 to 2008, the percentage of physicians who printed email communication and placed it in patients’ charts decreased from 48% (331/689) to 39% (159/408) (c^2^_1_ = 8.4, *P* = .004); the percentage of physicians who informed patients about privacy issues with respect to email decreased from 36.3% (250/689) to 29.2% (119/408) (c^2^_1_ = 5.7, *P* = .02); the percentage of physicians who notified patients to discuss emails when they become too lengthy decreased from 21.5% (148/689) to 15.7% (64/408) (c^2^_1_ = 5.5, *P* = .02); and the percentage of physicians who sent patients a message to inform them of completing a request decreased from 16.1% (111/689) to 11% (45/408) (c^2^_1_ = 5.4, *P* = .02).

**Table 3:** Physician’s self-reported adherence to recommended guideline items when emailing patients

	n (%) of Physicians		c^2^	DF^a^	*P*-value
Nationally recommended policies	2005 (n = 689)	2008 (n = 408)			
Print email communication and place inpatients’ charts	331 (48.0)	159 (39.0)	8.4	1	.004
Inform patients about privacy issues with respect to email	250 (36.3)	119 (29.2)	5.7	1	.02
When email messages become too lengthy, notify patients to come in to discuss or call them	148 (21.5)	64 (15.7)	5.5	1	.02
Establish a turnaround time for messages	111 (16.1)	53 (13.0)	1.9	1	.16
Request patients put their names or identification numbers in the body of the message	111 (16.1)	59 (14.5)	0.5	1	.75
Send a new message to inform patient of completion of request	11 (16.1)	45 (11.0)	5.4	1	.02
Establish types of transactions	11 (16.0)	64 (15.7)	0.0	1	.91
Explain to patients that their message should be concise	70 (10.2)	31 (7.6)	2.0	1	.16
Remind patients when they do not adhere to guidelines	55 (8.0)	30 (7.4)	0.1	1	.71
Develop archival and retrieval mechanisms	57 (8.3)	32 (7.9)	0.2	1	.74
Instruct patients to put category of transactions in subject line of message	48 (7.0)	21 (5.1)	1.4	1	.23
Configure automatic reply to acknowledge receipt of patients’ messages	42 (6.1)	20 (4.9)	0.7	1	.41
Request patients to use autoreply features to acknowledge clinician’s message	28 (4.1)	15 (3.7)	0.1	1	.47

^a^ DF: degrees of freedom.

### Predictors of Email Use in 2008

We investigated whether physician and practice characteristics among 2008 respondents were associated with email use with patients. Specifically, we present unadjusted and multivariate relationships between email use and gender, age, practice size, specialty, and practice setting in Table 4.

**Table 4:** Predictors of email use with patients among physicians in Florida (n = 1766)

		Physicians who used email with patients	Unadjusted odds ratio (95% CI)	Adjusted odds ratio^a^ (95% CI)
**Gender**				
	Male	274 (19.7%)	1.00	1.00
	Female	130 (21.9%)	1.14 (0.90-1.44)	1.48 (1.12-1.95)
**Age**				
	Less than 40 years old	78 (22.3%)	1.00	1.00
	41-50 years	133 (22.1%)	0.99 (0.72-1.36)	1.21 (0.85-1.71)
	51-60 years	131 (21.1%)	0.93 (0.68-1.28)	1.35 (0.94-1.94)
	61 years or older	62 (15.2%)	0.63 (0.43-0.90)	1.16 (0.76-1.79)
**Practice size**				
	Solo practice	122 (18.0%)	1.00	1.00
	2-9 physicians	199 (19.9%)	1.13 (0.88-1.46)	0.93 (0.71-1.23)
	10-49 physicians	51 (29.7%)	1.66 (1.14-2.41)	0.98 (0.61-1.56)
	50 or more physicians	29 (35.8%)	2.54 (1.55-4.16)	1.29 (0.68-2.43)
**Physicianspecialty**				
	Primary care	243(18.6%)	1.00	1.00
	Other	162 (23.7%)	1.36 (1.08-1.70)	1.43 (1.12-1.84)
**Practicesetting**				
	Single specialty	240 (17.5%)	1.00	1.00
	Multispecialty	133 (28.7%)	1.89 (1.48-2.41)	1.76 (1.30-2.37)
**Competency as a computer user**				
	Very sophisticated	74 (35.4%)	1.00	1.00
	Sophisticated	172 (25.0%)	0.61 (0.44-0.85)	0.55 (0.38-0.79)
	Neutral	137 (17.9%)	0.40 (0.28-0.56)	0.38 (0.26-0.55)
	Unsophisticated	23 (8.4%)	0.17 (0.10-0.28)	0.14 (0.08-0.26)
	Very unsophisticated	2 (4.9%)	0.94 (0.22-0.40)	0.10 (0.02-0.43)

^a^ Adjusted odds ratios control for all variables in the table.

In an unadjusted analysis of 2008 data, physician in the oldest age category (61 years or older) were significantly less likely to email with patients than those in the youngest category (OR 0.63, 95% CI 0.43-0.90). Moreover, as practice size increased, so did the tendency among respondents to indicate they used email with patients. For example, those in practices with 50 or greater physicians were significantly more likely than those in solo practices to email with patients (OR 2.54, 95% CI 1.55-4.16). Lastly, specialist physicians were more likely than primary care physicians (OR 1.36, 95% CI 1.08-1.70) and those in a multispecialty practice were more likely than those in a single specialty practice (OR 1.89, 95% CI 1.48-2.41), to email with patients.

In multivariate analyses of 2008 data that controlled for confounders, female physicians were more likely to indicate they email with their patients (OR 1.48, 95% CI 1.12-1.95). Additionally, specialist physicians were more likely than primary care physicians (OR 1.43, 95% CI 1.12-1.84) and those in a multispecialty practice were more likely than those in a single specialty practice to use email with their patients (OR 1.76, 95% CI 1.30-2.37). Self-reported computer competency levels among physicians predicted email use at every level of response. When compared with “very sophisticated” computer users, “sophisticated” users (OR 0.55, 95% CI 0.38-0.79), neutral users (adjusted OR 0.38, 95% CI 0.26-0.55), “unsophisticated” users (OR 0.14, 95% CI 0.08-0.26), and “very unsophisticated” users (OR 0.10, 95% CI 0.02-0.43) were all less likely to use email with patients. Lastly, in the multivariate analysis, physician age and practice size were no longer associated with email use with patients.

## Discussion

The benefits of email communication between physician and patient have been espoused by many researchers [1,5,6]. It has been reported that email between physician and patient can improve efficiency and workflow within a medical practice, and improve access to care and convenience to patients [1,5,6,28]. Despite the improvements this communication medium can provide, Florida physicians in 2005 were infrequently using emailing with patients [9]. In the current study, we made use of newly collected data from Florida to examine trends in email use by physicians over time.

The main finding of our analysis suggests that, while a higher percentage of physicians reported having tried emailing with patients in 2008 than in 2005, the proportion of physicians who are actively doing so on a regular basis did not change significantly during this time frame. Furthermore, physicians who had not yet tried emailing with patients had a waning future interest in doing so. On the contrary, relative to 2005, physician use of email from their practices with entities other than patients remained high. In fact, in 2008, physicians reported an increase in email use with individuals at hospitals and pharmaceutical companies, suggesting that physicians did see value in this communication medium with selected stakeholders.

A troubling trend involves the lack of adherence to professionally developed best practices designed to protect physicians who choose to email with patients. In 2005, we found that very few doctors abided by most of the recommended best practices developed by the AMA and AMIA. Our updated data from 2008 suggest that even fewer physicians who email with patients were adhering to these best practices. Specifically, even though a greater number of physicians reported abiding by at least one of the 13 guidelines, overall, fewer physicians reported adherence to all 13 guidelines with significant reductions in 4 guidelines. It is possible that physicians were not aware that these guidelines exist. Furthermore, it is possible that, despite their knowledge of these guidelines, physicians found it difficult to incorporate these best practices into their routine workflows. It is also possible that these guidelines may be perceived as outdated given that they were published in 1998 when email usage was much more infrequent between doctors and patients. Our belief is that the guidelines are still relevant and thus, given our findings, physicians are still exposing themselves to potentially unnecessary liability when they fail to heed the recommendations of the AMA and AMIA with respect to email use. Efforts should be made to draw attention to these guidelines, as well as simultaneously demonstrating how these guidelines can be adopted by physicians and integrated into their practice’s workflow. 

In 2008, several physician or practice characteristics were associated with email use with patients. In multivariate analyses, female physicians, specialty physicians (as opposed to primary care physicians), and those in a multispecialty practice were all more likely than their counterparts to use email with their patients. Given the increasing time demands on primary care physicians in terms of providing recommended services and preventative care [29], and the increasing length and volume of primary care visits [30], these physicians may have less time available to devote to emailing patients. This is particularly important in light of the national trend toward improving patient-centered medical homes in which primary care physicians are empowered to increase services, including through electronic means, to their patients.

On the other hand, physicians in multispecialty practices can gain economies of scale that help with certain administrative processes [31], which may provide more free time to use email with patients. Lastly, it is not clear why female physicians were more likely than their male counterparts to use email with patients. However, previous researchers have found that female doctors were more likely to earn continuing medical education (CME) credits online [32] and be responsive to email invitations to CME programs [33], both activities that may increase their proclivity to use information technology within their practice. More research is needed to more fully understand this trend. 

While, in 2005, physicians in the largest practices emailed with patients most frequently, practice size was no longer a significant predictor of email use in 2008. Furthermore, while age was negatively associated in univariate analysis with patient email use in both 2005 and 2008, multivariate analysis of 2008 data that included the newly available measure of self-reported computer sophistication made differences by age disappear. Our data show that computer sophistication may be a better predictor than age of technology adoption among physicians. Those who were very sophisticated computer users were significantly more likely to report emailing with patients than those who were sophisticated, neutral, unsophisticated, or very unsophisticated in increasingly higher proportions.

There are several limitations of this study worth mentioning. First, given that our data relied on self-reported information, we realize that our data are limited by participants’ ability to recall information accurately and their willingness to do so. Second, we recognize that response rates to both surveys were suboptimal. However, several researchers, including our group using the current data, have found that response bias in studies of health information technology are not likely, given the noncontroversial nature of questions on such surveys [34,35]. Third, given the pooled time-series nature of our analyses, we cannot be certain that the same physicians responded to our survey in 2005 and 2008. Even though we used very similar sampling methodologies, the characteristics of the 2005 and 2008 samples were different in some ways, including gender and specialty. Although these differences may be true changes in demographic characteristics among Florida physicians over time, we recognize that these differences may be a weakness of the study. Lastly, our study was conducted in a single US state where demographic, socioeconomic, and medicolegal characteristics affecting physicians may not generalize well to the rest of the country. Thus, we recommend caution when interpreting our results as reflective of physicians outside of Florida.

If physician-patient email communication is indeed valued as a patient-centered approach to improving health care quality, more effort will be needed to alleviate physician reluctance to engage in this activity. In Florida, the proportion of physicians who regularly email with patients rose only slightly between 2005 and 2008. This was a period when other health information technology applications such as electronic medical records and e-prescribing increased significantly in adoption nationally and in Florida [36,37]. Policy makers will need to seriously consider ways in which to encourage this activity if the potential benefits from physician-patient emailing are to be realized. Physicians who are using or are considering using email with patients are urged to become knowledgeable of best practices, which they can employ in their organizations.

## Multimedia Appendix 1

Survey of physician information technology use in Florida 2008, developed by Nir Menachemi, PhD and Robert G Brooks, MD

## Abbreviations

AMA: American Medical Association
AMIA: American Medical Informatics Association
CME: continuing medical education
