# Supplementary material for: The Use of Physician-Patient Email: A Follow-up Examination of Adoption and Best-Practice Adherence 2005-2008
Source: J Med Internet Res. 2011 Feb 25;13(1):e23. doi: 10.2196/jmir.1578 (PMC3221345; doi:10.2196/jmir.1578)
Supplement: Supplementary file 1 [file jmir_v13i1e23_app1.pdf]

Use of Information Technology by Physicians

1. Do you have internet access at your current office practice location?

☐ **YES** *If **YES**, please answer the following:*

What type of internet access do you have? *(Please ✓ all that apply)*

☐ Dial-up ☐ Wireless ☐ High Speed (i.e. cable or DSL)

☐ **NO**

2. How satisfied are you with the level of computerization in your current office practice?

☐ Very Satisfied ☐ Somewhat Satisfied ☐ Neutral ☐ Somewhat Dissatisfied ☐ Very Dissatisfied

3. Overall, how sophisticated of a computer user do you consider yourself?

☐ Very Sophisticated ☐ Sophisticated ☐ Neutral ☐ Unsophisticated ☐ Very Unsophisticated

4. Does your current office <u>practice</u> use <u>Electronic</u> <u>Health</u> <u>Records</u> (EHR)?

EHR is defined as a paperless form of the medical record that requires the provider to enter patient information (i.e., clinical notes) into a computer system instead of doing so on paper.

☐ **YES** *If **YES**, please answer the following:*

What <u>YEAR</u> best describes when you began using EHR in your practice? _______________

☐ **NO** *If **NO**, please answer the following:*

Are you considering getting EHR? *(Please ✓ one)*

☐ **YES**, very soon (within 1 year)
☐ **YES**, but not within the next year
☐ **NO**, I am not considering getting EHR at this time

5. If you <u>personally</u> routinely use Electronic Health Records (EHR) in your <u>office practice</u>, which of the following functions does your EHR include? ***(Please ✓ all that apply)***

☐ Problem list
☐ Procedures
☐ Diagnoses
☐ Medication list
☐ Allergies
☐ Patient demographics (i.e., age, DOB, etc.)
☐ Clinical notes
☐ Electronic prescribing of medications
☐ Electronic order entry (i.e., labs or x-rays)
☐ Electronically available lab data/ results
☐ Electronically available x-ray results
☐ Electronic connection to pharmacy info
☐ Patient scheduling
☐ Weight-based dosing calculations
☐ Growth charting
☐ Clinical decision support
☐ Patient education materials
☐ Coding advice to physicians
☐ Advance directives
☐ Access to reference material
☐ Preventive service reminders
☐ Auto-updated insurance coverage info
☐ Offsite access/ log-in capability
☐ Other (*Specify*):________________________

6. **Please indicate how each potential barrier affects your decision to continue (or expand) using EHR. <u>If you do not currently use EHR</u>, please respond by indicating how much each barrier contributes to why you don't currently use EHR in your office practice.**

	POTENTIAL BARRIERS			
**<u>Productivity</u>**	**Major Barrier**	**Minor Barrier**	**Not a Barrier**	**Not Applicable**
• Lack of time to acquire, implement such a system	☐	☐	☐	☐
• Entering data into computer can be cumbersome	☐	☐	☐	☐
• No time to learn how to use such a system	☐	☐	☐	☐
• The system would be difficult to use	☐	☐	☐	☐
• EHR may slow me down	☐	☐	☐	☐
• Disrupts workflow and/or office's physical layout to accommodate going to a computerized system	☐	☐	☐	☐
• Temporary loss of productivity and/or revenue during EHR system implementation phase	☐	☐	☐	☐
**<u>Financial</u>**				
• Inadequate Return on Investment (ROI)	☐	☐	☐	☐
• Upfront cost of hardware/software are too high	☐	☐	☐	☐
• Ongoing maintenance costs would be too high	☐	☐	☐	☐
**<u>Technical</u>**				
• Lack of uniform data standards within the industry	☐	☐	☐	☐
• Products available do not meet my needs	☐	☐	☐	☐
• Me and/or my staff don't have any technical knowledge	☐	☐	☐	☐
• Temporary loss of access to patient records if computer crashes or power fails	☐	☐	☐	☐
**<u>Patients</u>**				
• Privacy/confidentiality concerns (i.e., electronic records not secure)	☐	☐	☐	☐
• Patient resistance/ not wanting their physicians to use EHR	☐	☐	☐	☐

7. Does your current office practice use <u>electronic</u> <u>prescribing</u>?

E-prescribing is defined as use of an electronic system that enables physicians to send prescriptions and refill authorizations to any electronically enabled pharmacy's computer and also receive refill authorizations requests from the pharmacy

☐ **YES** *If **YES**, please answer the following:*

What <u>YEAR</u> best describes when you began using E-prescribing in your practice? ___________

What percentage of <u>NEW</u> prescriptions do you E-prescribe currently? ___________%

What percentage of <u>REFILL</u> authorizations do you E-prescribe currently? ___________%

What functions are included in your E-prescribing system? *(Please ✓ all that apply)*

- ☐ Drug list you previously prescribed for patient
- ☐ Drug list that others have prescribed for patient
- ☐ Formulary coverage look-up (through the e-prescribing system)
- ☐ Patient insurance coverage eligibility look-up (through e-prescribing system)
- ☐ Intelligent decision support tools (e.g., severe drug interaction alerts)

☐ **NO** **Are you considering electronic prescribing?** *(Please ✓ one)*

- ☐ **YES**, very soon (within 1 year)
- ☐ **YES**, but not within the next year
- ☐ **NO**, I am not considering getting E-prescribing at this time

8. Do you personally use email from your office practice to communicate with patients?

☐ **YES** *If **YES**, please answer the following:*

How often do you email patients?

☐ Often (at least once on ½ of all business days) ☐ Occasionally ☐ Rarely

Which of the following policies, if any, do you require for e-mail with your patients?

☐ Establish a turnaround time for messages
☐ Inform patients about privacy issues with respect to e-mail
☐ Print e-mail communications and place in patient's chart
☐ Establish types of transactions (i.e., prescription refill, appointment scheduling, etc.)
☐ Instruct patients to put category of transaction in subject line of message
☐ Request patients put their name or identification number in body of message
☐ Configure automatic reply to acknowledge receipt of patient's message
☐ Send a new message to inform patient of completion of request
☐ Request patients use auto-reply feature to acknowledge reading clinician's message
☐ Develop archival and retrieval mechanisms
☐ Explain to patients that their message should be concise
☐ Remind patients when they do not adhere to guidelines
☐ When e-mail messages become too lengthy, notify patients to come in to discuss or call them

☐ **NO I DON'T personally use email with patients.** *Please answer the following:*

Would you like to communicate with your patients by email in the future?

☐ Yes
☐ No
☐ Don't Know yet

9. Other than patients, do you use email from your practice with any other groups?

☐ **YES** *If **YES**, please answer the following:*

Which of the following groups do you use email with? *(Please ✓ all that apply)*

☐ Family member or caregiver of patients
☐ Other doctors
☐ Business related communications (e.g., with insurers, pharmacies, etc.)
☐ Hospitals
☐ Pharmaceutical companies
☐ My personal friends or family members
☐ Other (please specify):____________________________

☐ **NO**

DEMOGRAPHIC INFORMATION

10. Which of the following best describes the area in which you currently spend the majority of your practice time? (Please select only **one** choice)

- ☐ Family Medicine
- ☐ Internal Medicine
- ☐ Pediatrics
- ☐ OB/GYN
- ☐ General Surgery
- ☐ Surgical Specialty *(Specify)* ____________________
- ☐ Medical Specialty *(Specify)*____________________
- ☐ Pediatric Sub-specialty *(Specify)* ________________
- ☐ Other *(Specify)* ________________________________

11. Estimate the percent of your practice that is made up of patients in the following age groups:

0-18 years ______ %

19-64 years ______ %

65 years and over ______ %

12. Approximately what percentages of your patients have the following insurance coverage?

Medicare ______ %

Medicaid _____ %

Private insurance _____ %

Self-pay _____ %

Uninsured _____ %

13. How many physicians, including yourself, work at the practice location where you spend the majority of your time?

___________ # of physicians

14. Which single setting best describes where the majority of your time is spent?

- ☐ Single specialty private practice
- ☐ Multi specialty private practice
- ☐ Hospital or Emerg. Dept. (hospital employee)
- ☐ Hospital-owned office-based practice (hospital employee)
- ☐ Group or staff model HMO
- ☐ Academic health center/ university setting
- ☐ Community health center
- ☐ County health department
- ☐ Other *(Specify)* ____________________

15. How long have you practiced . . .

In your current community? __________ **YEARS**

Total years in practice (since medical school graduation) __________ **YEARS**

16. What is your gender? ☐ Male ☐ Female

17. What is your Age: ☐ Less than 40 years ☐ 41-50 years ☐ 51-60 years ☐ 61 years or greater

18. ☐ **Yes**, I would like to receive a summary of the findings. Email:___________________________

Thank you for your help!!!

Please return survey in the pre-addressed, postage-paid envelope to:
FSU Survey Research Laboratory Tallahassee, Florida 32306-2221
